# Genetic Background Effects on Disease Onset and Lifespan of the Mutant Dynactin p150^Glued^ Mouse Model of Motor Neuron Disease

**DOI:** 10.1371/journal.pone.0117848

**Published:** 2015-03-12

**Authors:** Terry D. Heiman-Patterson, Elizabeth P. Blankenhorn, Roger B. Sher, Juliann Jiang, Priscilla Welsh, Meredith C. Dixon, Jeremy I. Jeffrey, Philip Wong, Gregory A. Cox, Guillermo M. Alexander

**Affiliations:** 1 Department of Neurology, Drexel University College of Medicine, Philadelphia, Pennsylvania, United States of America; 2 Department of Microbiology Drexel University College of Medicine, Philadelphia, Pennsylvania, United States of America; 3 The Jackson Laboratory, Bar Harbour, Maine, United States of America; 4 Department of Neurology, Johns Hopkins University, Baltimore, Maryland, United States of America; 5 Department of Molecular and Biomedical Sciences, University of Maine, Orono, Maine, United States of America; National Institute of Health, UNITED STATES

## Abstract

Amyotrophic lateral sclerosis (ALS) is a neurodegenerative disease primarily affecting motor neurons in the central nervous system. Although most cases of ALS are sporadic, about 5–10% of cases are familial (FALS) with approximately 20% of FALS caused by mutations in the Cu/Zn superoxide dismutase (SOD1) gene. We have reported that hSOD1-G93A transgenic mice modeling this disease show a more severe phenotype when the transgene is bred on a pure SJL background and a milder phenotype when bred on a pure B6 background and that these phenotype differences link to a region on mouse Chromosome 17.To examine whether other models of motor neuron degeneration are affected by genetic background, we bred the mutant human dynactin p150^Glued^ (G59S-hDCTN1) transgene onto inbred SJL and B6 congenic lines. This model is based on an autosomal dominant lower motor neuron disease in humans linked to a mutation in the p150^Glued^ subunit of the dynactin complex. As seen in hSOD1-G93A mice, we observed a more severe phenotype with earlier disease onset (p<0.001) and decreased survival (p<0.00001) when the G59S-hDCTN1 transgene was bred onto the SJL background and delayed onset (p<0.0001) with increased survival (p<0.00001) when bred onto the B6 background. Furthermore, B6 mice with an SJL derived chromosome 17 interval previously shown to delay disease onset in hSOD1-G93A mice also showed delays onset in G59S-hDCTN1 mice suggesting that at least some genetic modifiers are shared. We have shown that genetic background influences phenotype in G59S-hDCTN1 mice, in part through a region of chromosome 17 similar to the G93-hSOD1 ALS mouse model. These results support the presence of genetic modifiers in both these models some of which may be shared. Identification of these modifiers will highlight intracellular pathways involved in motor neuron disease and provide new therapeutic targets that may be applicable to motor neuron degeneration.

## Introduction

Amyotrophic lateral sclerosis (ALS) is typically an adult-onset neurodegenerative disease affecting primarily the motor neurons in the spinal cord, brainstem, and motor cortex. Loss of motor neurons leads to progressive paralysis, atrophy of denervated muscles, and ultimately death usually within 2 to 5 years from diagnosis. Although most cases of ALS are sporadic (SALS), about 5–10% of ALS cases are familial (FALS)[1–3]. While SOD1 mutations were the first identified [4,5] accounting for 20% of FALS cases, subsequent studies have identified familial ALS associated with a number of other mutations. These include mutations in FUS, TDP43, alsin, dynactin, senataxin (SETX), synaptobrevin/vesicle-associated membrane protein associated protein B (VAPB), FIG4, optineurin, ubiquillin 2, and profilin (PFN1) as well as associations with the hexanucleotide repeat in the intronic region of the C9orf72 gene[6,7]. This repeat has also been identified in at least 7% of sporadic cases of ALS[8]. There is phenotypic heterogeneity observed with these mutations. For instance, in families carrying the SOD1 mutation, siblings carrying the same mutation can differ in disease expression with some family members carrying the mutant gene but not expressing disease. Furthermore, in families with the C9ORF hexanucleotide repeat, some carriers manifest with dementia, some with ALS and some with both ALS and dementia. This heterogeneity suggests disease modifying genetic factors[9]. In this regard, a modifier of disease onset in people has recently been linked to chromosome 1 whereas a susceptibility gene (CYP27A1) has been linked to sporadic ALS[10].

Identification of these mutations led to the development of a number of transgenic mouse models of ALS. The first was based on the hSOD1-G93A mutation. These mice demonstrate pathologic changes of the motor system and progressive weakness with shortened survival similar to the human disease [11]. Similar to families with SOD1 mutations where affected individuals show a variable phenotype[9], there are background-dependent differences in disease phenotype in transgenic mice that carry the mutated hSOD1-G93A transgene. Expression of the hSOD1-G93A transgene in ALR, NOD.Rag1KO, SJL/J (SJL) or C3H/HeJ backgrounds show a more severe phenotype whereas a milder phenotype is observed in C57BL/6J (B6), C57BL/10SnJ (B10), BALB/cJ and DBA/2J inbred strains compared to mice bred in the original mixed (B6/SJL) background[12,13]. As in humans with phenotypic variations, these background differences are likely due to genetic modifiers of disease severity. In this regard, we have identified a Chromosome 17 (Chr17) quantitative trait locus (QTL) with extension of disease onset and increased lifespan on animals carrying alleles derived from B6 or B10 at this locus [14].

One of the additional models of disease based on a different mutant protein is the mutant (G59S) human dynactin p150^Glued^ transgenic mouse (G59S-hDCTN1). This model is based on a slowly progressive autosomal dominant lower motor neuron variant of familial ALS in humans that is linked to a mutation in the p150^Glued^ subunit of the dynactin complex. Similar to people with dynactin mutations, this mouse demonstrates clinical and pathologic changes of motor neuron disease[15,16]. These mice develop spontaneous tremors between five and six months of age. Once near end-stage of the disease, the mice start to lose weight, stop grooming, and eventually become paralyzed[15]. The motor neuron degeneration in this mouse model is thought to be due to altered axonal transport and vesicle trafficking. As a result of the mutation there is disruption of the dynein/dynactin complex that produces the motor neuron disease phenotype.

The goal of this study is to examine whether the phenotype of the G59S-hDCTN1 mouse model of motor neuron degeneration, which has a defined mechanism of action that only partially overlaps with that described for the hSOD1-G93A mouse model, is also affected by genetic background. This would suggest that there are also genetic modifiers for this motor neuron diseases causing mutation. Once identified, these modifiers would be of great interest, especially if there were overlapping modifiers that affected models with differing mechanisms of neuronal dysfunction.

## Methods

### Mouse Colonies

We established a colony of mutant p150^Glued^ transgenic mice expressing a mutant form (G59S) of the human dynactin 1 (DCTN1) gene. The mice were originally produced by Dr. Philip C. Wong at The Johns Hopkins University School of Medicine by injecting the transgene containing the G59S substitution into fertilized eggs from C57BL6/SJL F1 hybrid mice [16]. The mice were then maintained on a mixed B6xSJL/F1 background[16]. From these mice, we developed two inbred strains (C57BL/6J and SJL/J) by backcrossing the original mixed B6/SJL mice to either pure C57BL/6J (B6) or SJL/J (SJL) for at least 5 generations. These procedures are the same as the ones we used to create incipient congenic C57BL/6J (B6) and SJL/J (SJL) mouse strains expressing the hSOD1-G93A transgene[12].

We also established a colony of mutant p150^Glued^ transgenic mice utilizing a B6.SJL interval specific mouse line previously derived in our lab. This interval specific mouse carries the SJL chromosome 17 interval from 16 to 53 MB within an otherwise pure B6 background. The parent line for the B6.SJL chromosome 17 congenic line was derived for a study of the role of T helper cells in a mouse model of multiple sclerosis using SJL/J (SJL) and B10.S/SgMcdJ (B10.S) mice from The Jackson Laboratory (Bar Harbor, ME)[17]. Several lines were selected for derivation into traditional congenic lines by marker-assisted selection[18]. One of these lines carried the SJL chromosome 17 interval from a position greater than 16.1 MB to a position less than 52.7 MB within an otherwise pure B10S background. This line was bread to C57BL6 (B6) mice to generate the B6.SJL interval specific line used in this study. The B6.SJL Chromosome 17 interval specific mice were bred with transgene positive males from our parent colony of congenic B6 mice carrying the G59S-hDCTN1transgene. This mating allowed us to create mice that were initially heterozygous at the Chr17 interval (B6/SJL) or homozygous for B6 derived alleles. Transgene-positive heterozygous (B6/S) males were then mated to heterozygous females and the offspring resulting in mice that were either B6/B6, B6/SJL, or SJL/SJL(SS) within the interval. The appropriate offspring were iteratively mated in order to obtain mice with SJL homozygous intervals. Resultant G59S-hDCTN1 transgene positive males with the SS interval were used to create a B6.SJL line of Dynactin p150^glued^ mice (B17S.G59S-hDCTN1). This is the same procedure that we used to create interval specific mice expressing the G93ASOD1 human transgene[14]. All of the different strains used in this study (B6, SJL, B6/SJL and B17S) were bred heterozygous (carry one copy) for the G59S-hDCTN1 transgene. Animals are monitored daily for overall health when the litter is changed and they are fed and given water. Once onset occurs they are monitored for survival along with daily care. All procedures used in this study were approved by the Drexel University Animal Care and Use Committee (IACUC).

### Disease Onset

Animals were evaluated weekly to judge for onset. Assessments included weights, splay, tremor and ladder behavior. Onset by weight was determined by the point at which maximum weight was reached and then began to decrease without increase. Onset for splay, ladder down and ladder up is judged on a scale from 0 to 3 (Table 1). For splay, 0 is a perfect leg splay resulting on raising the hind limbs while a 3 is the inability to spread hind legs at all. For ladder behavior the animal is placed at the base of the ladder and encouraged to climb up and then down the ladder. For ladder up a 0 refers to climbing up the ladder with no slippage, 1 indicates 1 or 2 slips going up or not alternating legs, 2 indicates slipping with a prolonged (>20 seconds) to climb up the ladder and 3 indicates the mouse falls down the ladder on attempting to climb up. For climbing down the ladder a 0 is normal descent with alternating hind legs and no slippage, a 1 indicates either one or two slips or not alternating hind limbs, a 2 indicates that the mouse slips down most of the ladder and does not alternate its’ hind limbs and a 3 indicates that the mouse falls down the ladder. Tremor is scored as 1 if present and 0 if absent. Onset is the first day that the animal scores a 1 for two consecutive assessments and never again scores a 0.

**Table 1:** Grading of Assessments.

Score	0	1	2	3
Splay	Normal, splays both hind limbs above horizontal	Splays hind limbs below horizontal	Partial ability to spread hind limbs	Inability to spread hind limbs
Ladder Up	Normal, ascent with alternating front legs and no Slippage	1 or 2 slips or not alternating steps	Slipping with a prolonged (>20 seconds) time to climb ladder	Mouse falls down the ladder when attempting to climb
Ladder Down	Normal, descent with alternating hind limbs and no slippage	1 or 2 slips and not alternating hind limbs	Mouse slips down the ladder and does not alternate hind limbs	Mouse falls down the ladder

### Survival

In this study, when an animal meets our euthanasia criteria, its age at sacrifice is considered the survival time. All efforts are taken to minimize pain and discomfort. Progressive deterioration of the animals' health leading to death was not allowed. Natural death was not an end point in this study. Mice are sacrificed by an overdose of barbiturates when they demonstrated limb paralysis or were unable to right themselves in 10 seconds when placed on their side.

### Genotyping

The mice were genotyped using DNA isolated from a 0.5 cm piece of mouse tail. Isolation was performed with the GenEluteTM mammalian genomic DNA miniprep kit (Sigma, St. Louis MO). The presence of the G59S-hDCTN1 mutant gene was determined by QPCR using iTaq universal SYBR Green supermix reagent (Bio-Rad, Hercules, CA). Briefly, 2.275 ng (6.5 μl of 0.35 ng/μl solution) of genomic DNA was added to a reaction mixture containing 12.5 μl of the universal supermix, 3 μl each (final concentration 0.3 μM) of the forward (5’ATGGGTGGGCGTGATTCTG-3’) and reverse (5’-ACTGGCGTACAAAGATGCCG-3’) primers for a total volume of 25 μl. After initial activation of the Taq polymerase at 95°C for 5 min, 40 PCR cycles of 95°C for 30 sec, 58°C for 30 sec and 72°C for 45 sec were performed. Assays were performed, in duplicate, on a Chromo 4 Quantitative PCR System (Bio-Rad, Hercules, CA). The B6.SJL Chromosome 17 interval specific mice were characterized by PCR using two markers (D17Mit51 and D17Mit176) located within the interval, that differ between SJL and B6.

### Statistics

Significance between groups was determined by analysis of variance (ANOVA) using the Tukey-Kramer post-hoc multiple comparison test. The data was considered significantly different if p< 0.05. Statistical calculations were accomplished with the aid of SYSTAT version 13 (SYSTAT Software Inc., Chicago, IL).

## Results

One hundred and forty five G59S-hDCTN1 mutant transgene-positive mice were used in this study. These mice consisted of 37, 48, 42 and 18 mice on the B6/SJL, B6, SJL and B17S backgrounds respectively. At the time of manuscript preparation all mice had developed motor neuron disease symptoms and 120 had met the euthanasia criteria and were sacrificed.

### Disease onset

**Fig 1:**
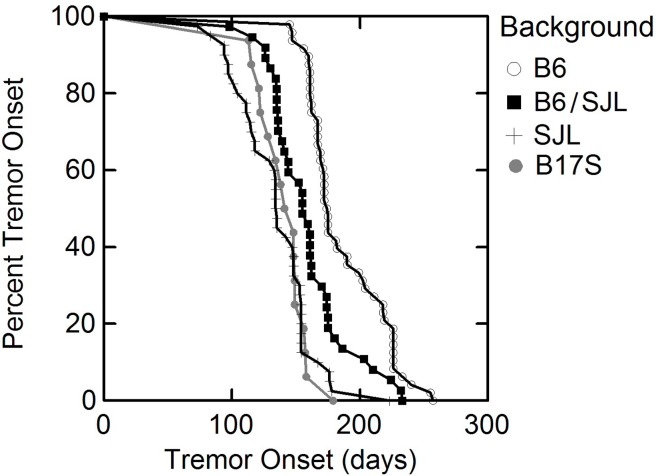
Onset is delayed when the G59S mutant dynactin transgene is placed on the B6 background while it is accelerated on either SJL or B6 mice carrying the chromosome 17 interval when compared to the mixed background the transgene was originally bred on (B6/SJL).

Disease onset (days ± standard deviation) as determined by splay, tremor and ladder down in male and female transgenic G59S-hDCTN1 mutant dynactin p150^Glued^ mice in different genetic backgrounds is tabulated in Table 2. The most consistent assessment for onset was tremor although in some cases splay was more sensitive in detecting onset at an earlier time point. Ladder up scores and weight loss were not good predictors of disease onset. We observed a significant (p<0.0005) acceleration of tremor onset (134.7 ± 29.3 days, N = 42 vs. 159.3 ± 31.2 days; N = 37) when mice carried the transgene on the SJL/J background compared to the mixed B6/SJL background. There was a significantly (p<0.00005) milder phenotype with delayed tremor onset (187.5 ± 30.3 days; N = 48 vs. 159.3 ± 31.2 days; N = 37) in mutant p150^Glued^ mice on the C57BL/6J background compared to animals in the original mixed B6/SJL background (Fig. 1). Similarly, for all other assessments (splay, and ladder down), the relative time of onset was significantly (p<0.0005) shortened when the G59S-hDCTN1 mutant p150^Glued^ transgene was bred on to the SJL background and significantly (p<0.00005) later when bred on the B6 background as compared to the mixed B6/SJL mice.

**Table 2:** Disease onset for mutant (G59S) human dynactin p150^Glued^ mice.

	**Onset (days) by Splay**			**Onset (days) by Tremor**			**Onset (days) by Ladder down**		
**Background**	**Female**	**Male**	**Total**	**Female**	**Male**	**Total**	**Female**	**Male**	**Total**
**B6 (N = 48, 18F,30M)**	168.7±52.8	179.1±36.5	**175.2±43.1●**	194.9±34.3	183.1±27.3	**187.5± 30.3**●	225.6±43.1	226.9±34.2	**226.4±37.3●**
**B6/SJL (N = 37, 15F,22M)**	124.4±19.8	136.5±57.8	**131.6±46.2**	154.5±29.4	162.6±32.7	**159.3± 31.2■**	169.9±30.3	175.6±47.3	**173.3±40.9**
**SJL (N = 42, 19F,23M)**	117.9±44.3	114.9±31.3	**116.3±37.3**	140.9±30.4	129.6±27.9	**134.7±29.3**	162.4±38.4	148.8±32.0	**154.9±35.3**
**B17S (N = 18, 6F, 12M)**	105.3±29.1	139.3±40.5	**128.0±39.8**	139.0±17.8	141.9±19.0	**141.0±18.1**	166.5±66.8	161.4±28.3	**163.6±46.4**

Disease onset in days ± the standard deviation of male and female transgenic mutant (G59S) human dynactin p150^Glued^ mice in different genetic backgrounds. There was no significant difference in disease onset (p>0.05) between male and female mice in any background. In the three methods for determining disease onset (splay, tremor and ladder down), mice in the B6 (●) background demonstrated significantly (p<0.0005) delayed onset as compared to mice expressing SJL derived genes (SJL, B6/SJL and B17S). In addition, for tremor onset, mice in the B6/SJL (■) background demonstrated significantly (p<0.001) delayed onset as compared to mice in the SJL background. There were no significant difference in disease onset between mice in the SJL background and interval specific congenic mice (B17S). These mice have a pure B6 background except for an SJL derived (chromosome 17 from 16 to 53cM MB) segment.

Disease onset in the chromosome 17 congenic B17S.G59S-hDCTN1 mice was significantly (p<0.0005) earlier when compared to the mice in the B6 background without the SJL interval. There was no significant difference in disease onset (p>0.05) between male and female mice in any background.

### Survival

**Fig 2:**
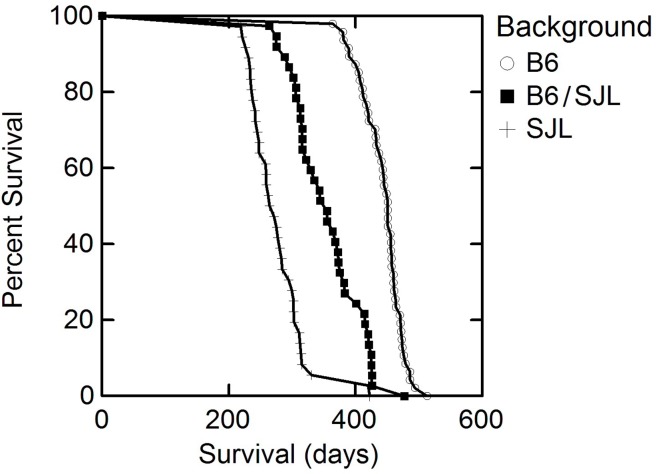
Survival is prolonged when the G59S mutant dynactin is placed on the B6 background and decreased when it is placed on the B6 background compared to the original mixed background (B6/SJL).

Survival (days ± standard deviation) of male and female transgenic mutant (G59S) human dynactin p150^Glued^ mice in different genetic backgrounds is shown in Table 3 and illustrated in Fig. 2. We observed shortened survival (276.6 ± 47.0 days; N = 36 vs. 355.0 ± 53.4 N = 37) when mutant p150^Glued^ was bred onto the SJL/J background as compared to the mixed B6/SJL colony (Table 3). There was an increased in survival (443.4 ± 32.7 days; N = 47 vs. 355.0 ± 53.4 N = 37) when mutant p150^Glued^ was bred on the C57BL/6J background compared to the original mixed B6/SJL background. All comparisons were statistically significant (p<0.0001).

**Table 3:** Survival for mutant (G59S) human dynactin p150^Glued^ mice.

	**Survival (days)**		
**Background**	**Female**	**Male**	**Total**
**B6 (N = 47; 17F, 30M)**	444.1 ± 37.8	443.0 ± 30.1	**443.4 ± 32.7**
**B6/SJL (N = 37; 15F, 22M)**	346.2 ± 54.9	361.0 ± 52.8	**355.0 ± 53.4**
**SJL (N = 36; 15F, 21M)**	274.4 ± 49.2	278.1 ± 46.5	**276.6 ± 47.0**

Survival in days ± the standard deviation of male and female transgenic mutant (G59S) human dynactin p150^Glued^ mice in different genetic backgrounds. There was no significant difference in survival (p>0.05) between male and female mice in any background. There were significant difference in survival between animals in the different backgrounds (B6 vs. B6/SJL p<0.00001; B6 vs. SJL p<0.00001; B6SJL vs. SJL p<0.00001).

The change in survival represented a 25% increase on the B6 background and a 28% shortening of lifespan on the SJL background when compared to the mixed parental B6/SJL background. Furthermore, there is a 60% difference, when lifespan was compared between the B6 and SJL backgrounds. This difference in lifespan is twice that of the G93ASOD1when bred onto a B6 background compared to the more rapid progressing NOD or SJL[13]. These differences in both onset and survival were significant (p<.001) for all comparisons across backgrounds. As with disease onset; there was no significant difference (p>0.05) in survival between males and females on any background.

All of the B17S.G59S-hDCTN1 animals show accelerated disease onset (Fig. 1, Table 2), are still alive and many are over 475 days old. The data shows that as with the G93A hSOD1 mice, the SJL derived 16–52 Mb chr 17 segment accelerates disease onset in G59S-hDCTN1 mutant mice and not survival given that these animals are alive well past the expected survival time for G59S-hDCTN1 bred on the pure SJL or the mixed B6/SJL background.

## Discussion

This is the first study to demonstrate background-dependent differences in phenotype in the G59S-hDCTN1 mouse model of motor neuron disease. This study showed that the progression of motor neuron disease and the survival in mutant G59S-hDCTN1 mice depend on genetic background with a more severe phenotype in SJL mice and a milder phenotype in B6 mice. The importance of this finding, given that there is no a priori reason for different strains of mice that carry the same mutant gene to demonstrate such different phenotypes, is that it strongly suggests that there are modifier genes that differ between strains that either accelerate or more importantly, slow the progression of disease.

The genetic background influences on disease phenotype in G59S-hDCTN1 transgenic mice was similar yet more pronounced than the phenotype differences we previously reported in the G93ASOD1 mouse[12,13]. Mice expressing the G59S-hDCTN1 transgene in the B6 background showed delayed disease onset and slower disease progression resulting in a 60% increase in lifespan (from 277 to 443 days) as compared to mice expressing the transgene in the SJL background; whereas lifespan is increased by 21% (from 119 to 144 days) when the G93ASOD1 transgene is placed on the B6 as compared to the SJL background. The disease phenotype of mice in the mixed B6/SJL background was intermediate between the two pure congenic lines for both transgenic types.

These findings are consistent with observations in human ALS where there is variation in the phenotype between siblings who both carry the same mutation[9]. Although the cause of this phenotypic variation has not been determined, it is likely related to genetic modifiers of disease expression inherent in the genetic heterogeneity even within members of the same family[2,6,9].

As further evidence that these background differences are due to genetic modifiers we have identified a Chromosome 17 QTL in G93A-hSOD1 mice that significantly extends the lifespan of animals carrying alleles derived from B6 or B10 at this locus[14]. In this regard we have reported that interval-specific congenic mice that carry a chromosome 17 interval (proximal 16 to 53 Mb) derived from SJL on an otherwise pure B6 background (B6.SJL-Chr17) accelerates onset in the transgenic G93A-hSOD1 mouse model of ALS from a B6 phenotype to an SJL phenotype confirming the presence of modifiers in this region. Here we report that this SJL derived interval also accelerates disease onset in mice expressing the G59S-hDCTN1 mutant gene. This suggests that some of the genetic modifiers are shared by these two models despite the fact that the motor neuron degeneration is caused by mutations in different proteins.

The G59S-hDCTN1 mutant mouse model is based on a slowly progressive autosomal dominant lower motor neuron disease in humans that is linked to a mutation in the p150^Glued^ subunit of the dynactin complex that demonstrates clinical and pathologic changes of motor neuron disease[15,16,19]. This mutation occurs in a highly conserved CAP-Gly motif of the p150^Glued^ subunit of dynactin that binds to microtubules[20].The dynactin complex is necessary for dynein mediated retrograde transport of vesicles and organelles along microtubules providing a link between the cargo, microtubules, and cytoplasmic dynein during transport of vesicles. The p150^Glued^ subunit promotes initiation of the dynein-driven retrograde transport likely by stabilizing the microtubule track[21,22]. Mutations in the p150^Glued^ subunit of dynactin likely cause disease by the disruption of retrograde transport and the destabilization of microtubules. While disease in the G93-hSOD1 mutant mouse has been attributed to a toxic gain of function of SOD1, the actual mechanism by which this toxic gain of function leads to upper and lower motor neuron dysfunction is unclear. Multiple mechanisms appear to be contributing to disease including oxidative stress, protein aggregation, mitochondrial defects, and axonal transport[23,24,25,26]. While some of these mechanisms are different than the G59S-hDCTN1 mouse, others including abnormalities of transport are shared[16,23,27], suggesting that any genetic modifiers of phenotype shared by these mouse models may be directed at either overlapping mechanisms or diverse mechanisms with convergent final pathways.

The findings of this study demonstrate that there are at least two regions of the mouse genome that modify the ALS phenotype in the G59S-hDCTN1 mouse. Some genetic modifiers of disease onset are located within the 16–53 MB proximal segment of chromosome 17 whereas modifiers of survival are located elsewhere. In order to extend these findings, we are conducting reciprocal backcrosses between G59S-hDCTN1 mutant B6 (mild phenotype) and SJL (severe phenotype) congenic mice in order to identify major quantitative trait loci (QTLs) that modify survival and onset of motor neuron disease caused by the dynactin p150^Glued^ (G59S-hDCTN1) mutation. In order to determine if, as previously shown in the hSOD1-G93A mouse model of ALS[14], genetic modifiers of survival are located in the proximal region of chromosome 17, we are developing G59S-hDCTN1 mutant mice on an interval-specific congenic that carries a longer segment of chromosome 17 from NOD mice. NOD mice like SJL exhibit an accelerated disease phenotype when expressing the G93A-hSOD1 mutant gene[14]. Interval specific congenic mice with the NOD interval from the proximal 1.0–70 MB of Chr17 on an otherwise B6 background are available from our collaborators at Jackson Laboratories.

In conclusion, this study demonstrates that in addition to the G93A-hSOD1 mutant mouse model, genetic background influences phenotype in a second mouse model of motor neuron disease, the G59S-hDCTN1 mutant. These results suggest that there are genetic modifiers in both these models and that some modifiers may be shared despite the differences in the underlying dominant mutations in proteins initiating disease. Identification of modifier genes, in particular modifier genes shared by different models, will highlight intracellular pathways involved in motor neuron disease and provide new therapeutic targets that may be applicable to motor neuron degeneration from multiple etiologies.

## Supporting Information

S1 Dataset Supporting Data. (XLSX)
